# Genome-wide detection of selection signatures in Jianli pigs reveals novel cis-regulatory haplotype in *EDNRB* associated with two-end black coat color

**DOI:** 10.1186/s12864-023-09943-9

**Published:** 2024-01-02

**Authors:** Zhong Xu, Junjing Wu, Yu Zhang, Mu Qiao, Jiawei Zhou, Yue Feng, Zipeng Li, Hua Sun, Ruiyi Lin, Zhongxu Song, Haizhong Zhao, Lianghua Li, Nanqi Chen, Yujie Li, Favour Oluwapelumi Oyelami, Xianwen Peng, Shuqi Mei

**Affiliations:** 1 Hubei Key Laboratory of Animal Embryo and Molecular Breeding, Institute of Animal Husbandry and Veterinary, Hubei Provincial Academy of Agricultural Sciences, Wuhan, 430064 China; 2 (College of Animal Sciences, College of Bee Science), Fujian Agriculture and Forestry University, Fuzhou, 350002 China; 3 The John Curtin School of Medical Research, Australian National University, Canberra, 2600 Australia; 4 Hubei Hongshan Laboratory, Wuhan, 430064 China

**Keywords:** Jianli pig, Whole-genome sequencing, Genetic diversity, Selection signatures, Coat color

## Abstract

**Background:**

Jianli pig, a renowned indigenous breed in China, has the characteristics of a two-end black (TEB) coat color, excellent meat quality, strong adaptability and increased prolificacy. However, there is limited information available regarding the genetic diversity, population structure and genomic regions under selection of Jianli pig. On the other hand, the genetic mechanism of TEB coat color has remained largely unknown.

**Results:**

In this study, the whole genome resequencing of 30 Jianli pigs within a context of 153 individuals representing 13 diverse breeds was performed. The population structure analysis revealed that Jianli pigs have close genetic relationships with the Tongcheng pig breed, their geographical neighbors. Three methods (observed heterozygosity, expected heterozygosity, and runs of homozygosity) implied a relatively high level of genetic diversity and, a low inbreeding coefficient in Jianli compared with other pigs. We used Fst and XP-EHH to detect the selection signatures in Jianli pigs compared with Asian wild boar. A total of 451 candidate genes influencing meat quality (*CREBBP*, *ADCY9*, *EEPD1* and *HDAC9*), reproduction (*ESR1* and *FANCA*), and coat color (*EDNRB*, *MITF* and *MC1R*), were detected by gene annotation analysis. Finally, to fine-map the genomic region for the two-end black (TEB) coat color phenotype in Jianli pigs, we performed three signature selection methods between the TEB coat color and no-TEB coat color pig breeds. The current study, further confirmed that the *EDNRB* gene is a candidate gene for TEB color phenotype found in Chinese pigs, including Jinhua pigs, and the haplotype harboring 25 SNPs in the *EDNRB* gene may promote the formation of TEB coat color. Further ATAC-seq and luciferase reporter assays of these regions suggest that the 25-SNPs region was a strong candidate causative mutation that regulates the TEB coat color phenotype by altering enhancer function.

**Conclusion:**

Our results advanced the understanding of the genetic mechanism behind artificial selection, and provided further resources for the protection and breeding improvement of Jianli pigs.

**Supplementary Information:**

The online version contains supplementary material available at 10.1186/s12864-023-09943-9.

## Background

The domestication of farm animals was a remarkable event that had a profound impact on human history [1]. The pig (Sus scrofa) was an economically important animal that was domesticated about 10,000 years ago [2]. Throughout a long history of evolution and breeding, pigs have been naturally or artificially selected for specific traits, such as fitness, coat color, meat quality, and so on. Therefore, detecting the genetic footprints left on the genome during domestication through genome sequencing is helpful in analyzing the origin and evolution of different breeds and the formation mechanism of important economic traits.

Jianli pig, one of the well-known indigenous breeds in China, was originally distributed in Jianli County, Hubei Province of China [3]. It has the characteristics of early maturity, good meat quality and strong disease resistance. Due to their superior meat quality, Jianli pigs were sent to the capital of the Qing Dynasty as tribute in the 16th year of Qianlong (1752) according to the records of Jianli County [3]. Sadly, the Jianli pig population has been declining over the past two decades due to the large number of Western pig breeds imported to increase the lean meat percentage of pork. For this reason, Jianli pigs have been included in the conservation list of China’s livestock and poultry genetic resource by the Ministry of Agriculture of China. On the other hand, Jianli pigs and Tongcheng pigs, are both located in the southern of Hubei Province and have the same coat color, but the genetic distance between them is still unclear. To better protect Jianli pigs, it is necessary to use genome-wide markers to study their genetic diversity and population structure.

Additionally, coat color phenotypes are important features for breed identification in livestock production. Jianli pig’s body and limbs are white, while its head, neck, rump, and tail are black, which is typical of a two-end-black (TEB) colored breed. Interestingly, studies on the genetic mechanisms behind the TEB coat color remain limited. For example, Wang et al. found that the *MITF* and *EDNRB* genes may influence the TEB color trait in Tongcheng pigs, a finding which however does not extend to Jinhua pigs [4]. Huang et al. [1] provided further evidence that the *EDNRB* is the gene responsible for the TEB phenotype in Chinese indigenous pigs, with the exception of the Jinhua pigs. However, Zheng et al. [5] found that, in Jinhua pigs, the TEB coat color was most likely influenced by the *EDNRB* gene, but through genetic mechanisms distinct from other Chinese TEB pig breeds. Until now, the genetic mechanism of TEB coat color has remained largely unknown.

In this study, we used whole-genome resequencing data of Jianli pigs in a context of 153 individuals from 13 diverse breeds to investigate the genetic diversity, signatures of selection in Jianli pigs. Further, we used signatures of selection, ATAC-Seq and luciferase assay to identified the regions shaped by selection for TEB coat color in Chinese indigenous pigs. Our findings enable us to better understand the Jianli pig’s genome characteristics, and provide novel insights for developing breeding strategies and germplasm conservation in the near future.

## Methods

### Sample collection and sequencing

Ear tissue was collected from 30 Jianli pigs, 12 Jinhua pigs and 7 Hubei white pigs for high-throughput resequencing in this study. Genomic DNA was extracted from pig ear tissue using the Qiagen DNeasy Tissue Kit (Qiagen, Germany) following a standard phenol-chloroform extraction procedure. Paired-end (2 × 150 bp) sequencing libraries were constructed for each individual and sequenced (~ 10 coverage) on the Illumina HiSeq 2000 platform (Novogene, China). Whole-genome sequencing (WGS) data of 104 individuals, including eight Chinese indigenous breeds, one Asian wild boar population and three European modern breeds (Table 1), were downloaded from public databases with project number PRJNA213179 [6], PRJNA488960 [7], PRJNA524263 [8], and PRJNA260763 [9]. In summary, whole genome sequencing data of 153 pig samples of 13 breeds were analyzed in this study.

### Variant calling and annotation

The paired-end reads were mapped to the reference genome Sscrofa11.1 using Burrows-Wheeler Aligner (BWA) v0.7.12 [10] with default settings and sorted binary bam files were obtained via SAMtools v1.9 [11]. Genome Analysis Toolkit (GATK) v4.1.4.1 [12] was used for SNP calling. The gVCF files were generated using the “HaplotypeCaller” module, and the joint genotypes were determined using “GenotypeGVCFs” module. Then, hard filtering was implemented under the criteria of QUAL < 30.0 || QD < 2.0 || FS > 60.0 || MQ < 40.0 || SOR > 3.0 || MQRankSum < -12.5 || ReadPosRankSum < -8.0 via the VariantFiltration method from GATK. Finally, a total of 25.16 million SNPs with minor allele frequency > 0.05 and call rate > 0.9 were obtained using vcftools v0.1.15 [13].

### Population structure analysis

The identical-by-state (IBS) and genetic differentiation (Fst) matrix calculated by PLINK v1.9 [14] were used to construct genetic distances between individuals and populations, respectively. The neighbor-joining (NJ) tree was built based on IBS and Fst matrix using the MEGA v5.0 [15] and visualized by FigTree v1.4.3 (http://tree.bio.ed.ac.uk/software/figtree/). Principal component analysis (PCA) was conducted with GCTA v1.91.7 software [16] and the first two principal components (PC) were plotted using in-house R scripts. Population structure analysis was carried out using ADMIXTURE v1.3 [17] with kinship (K) set from 2 to 11.

### Genetic diversity statistics

Observed heterozygosity (Ho) and expected heterozygosity (He) were calculated using PLINK with default setting to evaluate the genetic diversity. The level of runs of homozygosity (ROH) was estimated using PLINK v1.9 with the following parameters [18]: (1) a sliding window of 50 SNPs; (2) one heterozygous and five missing calls were allowed per window to account for genotyping error; (3) the minimum number of consecutive SNPs included in a ROH was set to 100; (4) to exclude short ROH that was derived from strong LD, the minimum length for a ROH was set to 1 Mb. The inbreeding coefficient (F_ROH_) was calculated as the sum length of ROH divided by the length of the autosomal genome covered by the SNPs.

### Detection of selective sweeps

To identify the genomic selection signatures of Jianli pigs, we calculated the averaged Fst and XP-EHH values of Jianli pigs and Asian wild pigs using vcftools and selscan [19] software with 40 kb sliding windows of 20 kb step, respectively. Extremely high values in the 3% right-tail of each method were empirically selected as potential candidate regions under positive selection. In order to reveal the genomic selection signatures of two-end‐black (TEB) coat color phenotype in Jianli pigs, we performed three methods including Fst, XP-EHH and locus‐specific branch length (LSBL). Fst and XP-EHH values were estimated between the TEB coat color pig breeds (Jianli, Jinhua, Luchuan, Bamaxiang, and Tongcheng, n = 64) and no-TEB coat color breeds (Asian wild boar, Erhualian, Laiwu, Wannan Black, Hubei White, Duroc, Landrace and Large White, n = 89). LSBL x for population A between populations B and C were calculated using pairwise Fst, where x = (FstAB + FstAC − FstBC)/2 and A, B, and C are the three populations under consideration [20]. Population A included five TEB‐colored breeds (Jianli, Jinhua, Luchuan, Bamaxiang, and Tongcheng, n = 64); (b) population B comprised four breeds with a solid black coat color phenotype (Asian wild boar, Erhualian, Laiwu, Wannan Black, n = 46); (c) population C included three breeds with a solid white coat color phenotype (Hubei White, Landrace and Large White, n = 36).

### Analysis of *EDNRB* haplotypes

A 1.9-kb region (SSC11: 50,086,427 − 50,088,340 bp) harboring 36 SNPs at *EDNRB* gene locus was phased using BEAGLE [21]. The phased haplotypes were explored to construct a haplotype‐sharing heat map using the pheatmap package in the R language. LDBlockShow software [22] was used with default parameters for linkage disequilibrium analysis and constructed haplotype blocks between different pig breeds from a single vcf files. The phased haplotypes were used to construct a haplotype network using the PopART 1.7 software [23].

### ATAC-seq analysis

To detect whether the haplotype is located in the functional regulatory region, we used publicly available ATAC-seq data from different tissues. The ATAC-seq data was downloaded from PRJEB44468. Fastp (v0.19.11) software was used to remove adapters and low-quality sequences to obtain high-quality clean reads. The sequences were mapped to the reference genome (Sus scrofa 11.1) using BWA v0.7.12 software. Samtools was used to convert SAM files to BAM format and for peak calls. Peak calls were made using MACS2 v2.1.2 software to get an overview of open chromatin regions. When the Q value is < 0.05, the region is defined as the peak. The peak distribution of different genomic regions was evaluated using ChIPseeker v1.16.1. Use the Integrated Genomic Viewer to view all sequencing trails and bigWig files.

### Luciferase reporter assay

The 25 SNPs found in the 2nd intron region of *EDNRB* were located in the functional regulatory region. In order to verify its function, we conducted dual-luciferase expression assays. To generate luciferase reporter constructs for the 25-SNPs haplotype of the *EDNRB* gene, we cloned the fragments of the Jianli and Xidu black *EDNRB* gene, respectively, into the pGL3-promoter (Promega, USA) vector. Then transfect the pGL3-promoter vectors and pRL-TK vector into PK-15 cells by Lipofectamine 3000 kit (Invitrogen, Carlsbad, CA, USA). Following the protocol of a dual-luciferase reporter assay system (Promega, Madison, WI, Germany) to quantify luciferase activity, it was calculated as the firefly fluorescence value / renilla fluorescence value (n = 3). Statistical analysis was conducted using a Student’s t-test, and a p-value lower than 0.05 was considered significant.

## Results

### Sequencing and variant detection

We performed whole-genome sequencing of 30 Jianli pigs, 12 Jinhua pigs and 7 Hubei White pigs with an average depth of 10×. To investigate the genetic diversity and population structure of Jianli pigs from a global perspective, we downloaded sequencing data for 104 individuals, including eight Chinese indigenous breeds, one Asian wild boar population and three European modern breeds (Table 1). Afterwards, we conducted variant detection in these 153 pigs and identified a total of 25.16 million SNPs, 20% of which were identified as novel based on their absence in the pig dbSNP database (ftp://ftp.ncbi.nih.gov/snp/organisms/pig9823/VCF/). In agreement with previous studies, most of the SNPs were located in intergenic and intron regions (58.6 and 39.8% respectively), while only 0.64% of them were located in exonic regions, including 248,528 synonymous and 82,508 nonsynonymous mutations.

**Table 1:** Samples and their genomic diversity statistics

Breed	Abbrev	Number	Ho	He
Jianli	JL	30	0.215311	0.226628
Jinhua	JH	12	0.183613	0.192367
Erhualian	EHL	5	0.221241	0.207351
Luchuan	LC	6	0.211148	0.190989
Bamaxiang	BMX	6	0.247661	0.226151
Laiwu	LWU	6	0.225258	0.217105
Tongcheng	TC	10	0.199808	0.227419
Wannan Black	WNH	20	0.188564	0.219435
Asian wild boar	AWB	15	0.217459	0.269978
Hubei White	HBBZ	7	0.168966	0.190005
Duroc	DU	8	0.113934	0.121821
Landrace	LR	13	0.146769	0.166592
Large White	LW	15	0.152152	0.171055

### Population structure analysis

**Fig. 1:**
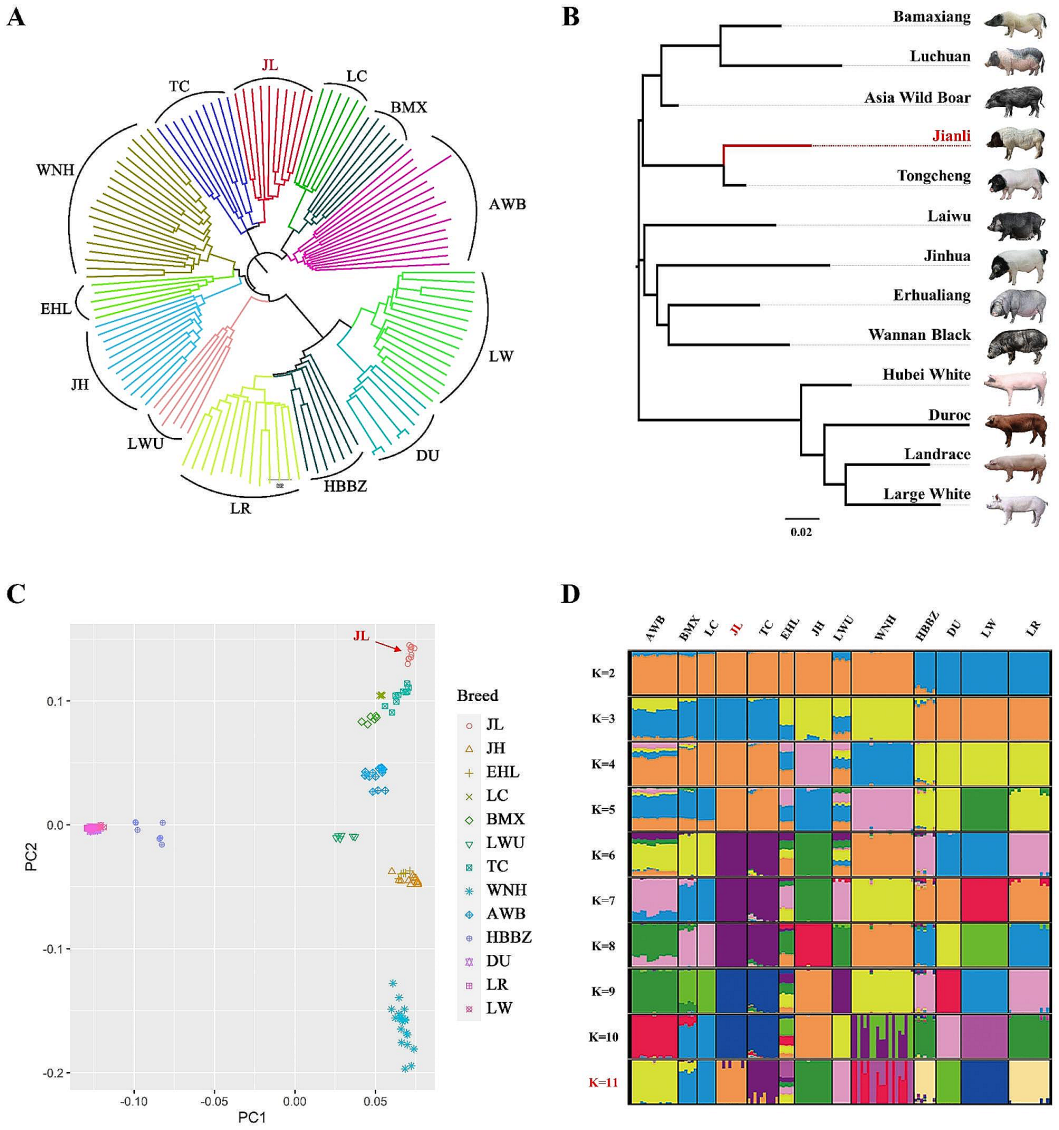
Population relationship and structure of Jianli pigs and other breeds tested in this study. **A**: NJ tree constructed by identity-by-state matrix among 153 samples. **B**: NJ tree based on pairwise FST values among 13 populations. **C**: Principle component analysis of 153 pigs using their first two components. **D**: Population structure of 13 pig breeds revealed by ADMIXTURE analysis (K = 2 to 11). Abbreviations for breeds are given in Table 1

To explore the phylogenetic relationships among Jianli pigs and other breeds, we conducted neighbor-joining (NJ) tree, principal component analysis (PCA) and ADMIXTURE using genomic SNPs. In a NJ tree between individuals (Fig. 1A) and populations (Fig. 1B), individuals from the same breed were clustered together, and then subdivided into two main branches representing Chinese indigenous breeds and modern commercial breeds. The result of the PCA analysis was consistent with the above NJ clustering pattern (Fig. 1C). It became evident that the Jianli pig shares the closest genetic relationships to the Tongcheng pig breeds. Further, to investigate admixture levels among all tested breeds, we performed the ADMIXTURE analysis assuming ancestral number K from 2 to 11 (Fig. 1D). When K = 2, two basal lineages represented modern commercial breeds and Chinese indigenous pigs, respectively. When K = 11, the Jianli and Tongcheng pigs formed an independent lineage while the Luchuan and Bamaxiang pigs were still together (Fig. 1D).

### Genetic diversity analysis

To further assess the genetic diversity of Jianli pigs in the context of global pig breeds, we estimated the expected heterozygosity (He), observed heterozygosity (Ho), inbreeding coefficient, and ROHs of each pig group. The range of expected He and Ho were 0.12–0.26 and 0.11–0.24, respectively (Table 1). In most groups, the He value was always observed to be higher than the Ho value, which indicates that inbreeding can occur in groups. Jianli pigs had Ho of 0.21 and He of 0.22, which were comparable with those of most Chinese indigenous breeds.

**Fig. 2:**
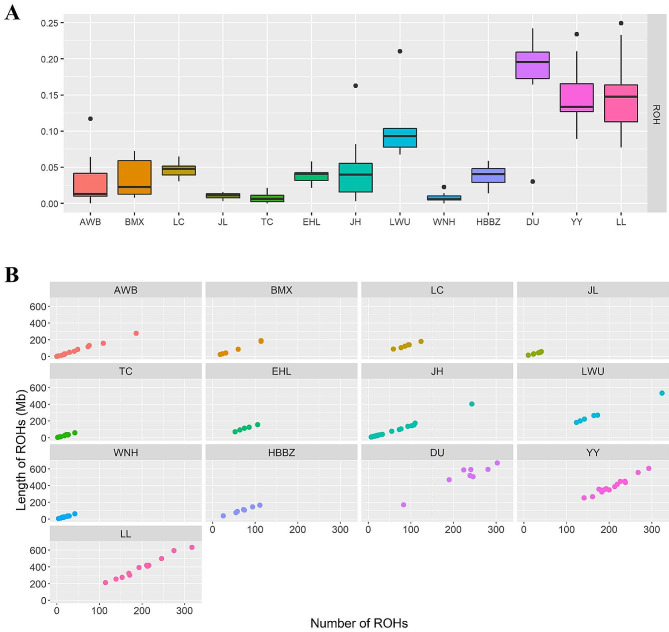
Genome-wide scan for ROH. **A**: Box plot of the inbreeding coefficients based on ROH (F_ROH_) for pig breeds. **B**: Number of ROH per breed (X-axis) and the total ROH length of each animal (Y-axis)

To evaluate the effect of inbreeding on the pig genome, we assessed the genome-wide autozygosities as ROH (Fig. 2A). We found that the inbreeding coefficient based on ROH varied both within and across breeds. Specifically, Duroc breed was characterized by the highest inbreeding (F_ROH_ = 0.199), followed by Landrace (F_ROH_ = 0.142), whereas WNH showed the lowest value (F_ROH_ = 0.007). Our results indicated that, Western pigs had a higher number of ROH than Chinese pig populations (Fig. 2B, Table S1). In agreement with previous studies, the modern breeds in general had longer runs of homozygosity than Chinese indigenous breeds. The Jianli pigs have similar heterozygosity (He and Ho), lower fragment length and lower inbreeding coefficient compared with other Chinese indigenous pigs.

### Selection signature detection of the Jianli pig population

**Fig. 3:**
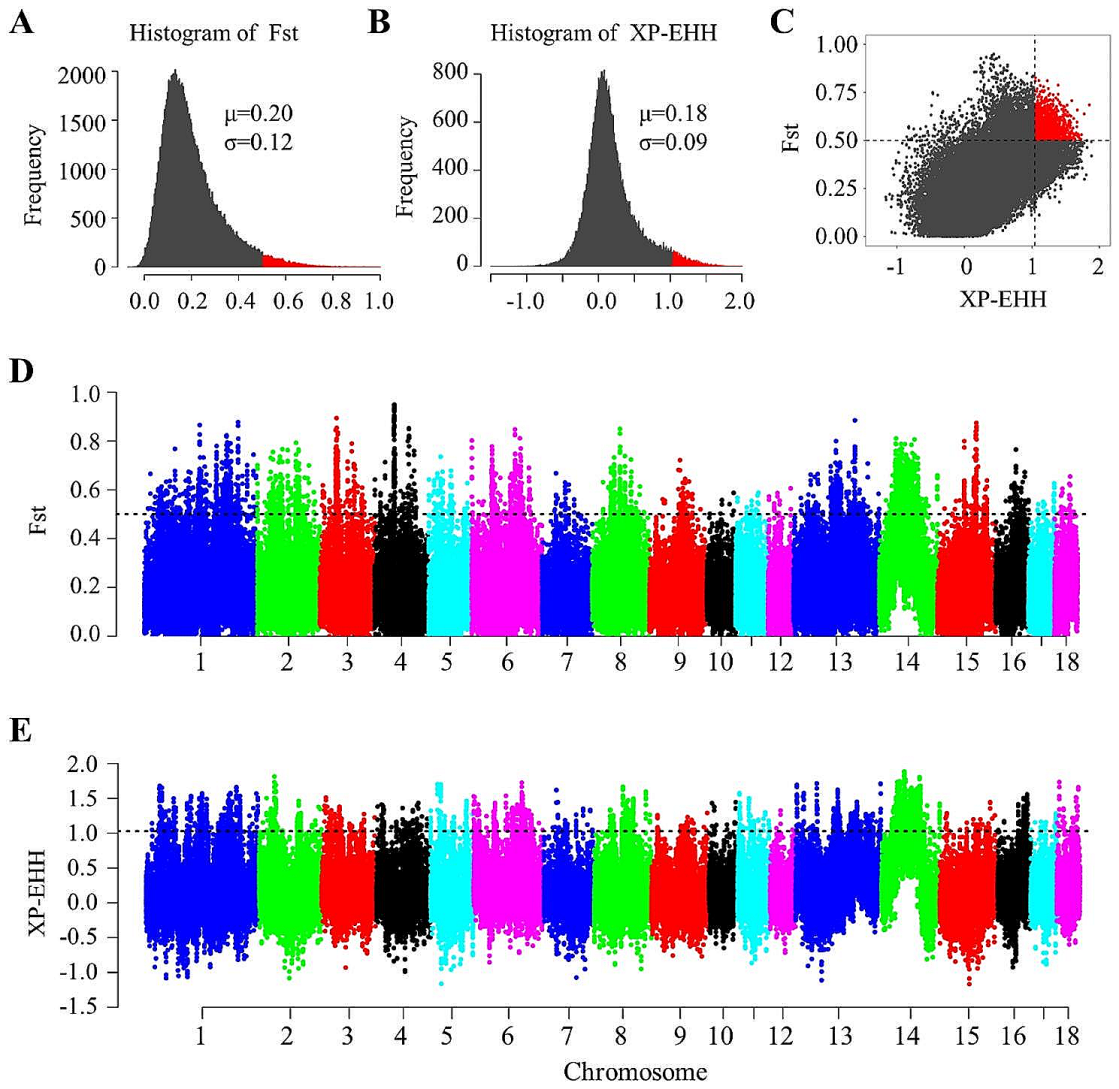
Genome-wide selection analysis of Jianli pigs. **A**: Histograms of the 40-kb windowed fixation index (Fst) of the autosomes. **B**: Histograms of the 40-kb windowed XP-EHH of the autosomes. **C**: The intersection of the two methods used to identify high-quality selection regions. **D**: Genome-wide distribution of selection signatures detected by Fst across each chromosome in Jianli and Asian wild boar pigs. **E**: Genome-wide distribution of selection signatures detected by XP-EHH across each chromosome in Jianli and Asian wild boar pigs

To explore the genomic evidence related to the breed features of Jianli pigs, we further compared the genomic signatures of Jianli pigs with Asian wild boar pig breed at the population level. We applied the Fst and XP-EHH methods to detect genomic regions related to selection in the Jianli pig breed. To minimize false-positive candidate regions, windows that simultaneously exhibited significantly high Fst values (in the top 3% of the right tail, where Fst is 0.50) and significantly high XP-EHH values (in the top 3% of the right tail, where XP-EHH is 1.03) were considered as the candidate selective regions in Jianli pigs (Fig. 3A, 3B and 3C). The genome distributions of candidate regions detected by different methods of Jianli pigs were shown in Fig. 3D and 3E. By applying these standards, we identified 3396 regions on autosomes with high Fst values (Table S2) and 3396 genomic regions with high XP-EHH values (Table S3). After merging neighboring windows into the selected regions, 382 putative selective regions with a total length of 28.44 Mb were identified in autosomes, accounting for 1.17% of the entire genome (Table S4).

A total of 451 genes were identified in the selected regions (Table S5), and their KEGG pathway analysis and Gene Ontology terms were performed by Metascape (http://metascape.org). Most of the genes were involved in immunoglobulin production (GO:0002377), melanocyte differentiation (GO:0030318), fibroblast proliferation (GO:0048144) and male infertility pathway (WP4673) (Figure. S1). Several candidate genes relate to coat color trait, such as *EDNRB*, *MITF*, and *MC1R*. Some genes related to meat quality were detected: *CREBBP, ADCY9, EEPD1*, and *HDAC9*. Some genes associated with reproduction traits were identified, such as ESR1 and FANCA.

### Detection of candidate genes associated with two-end black coat color of Jianli Pig

**Fig. 4:**
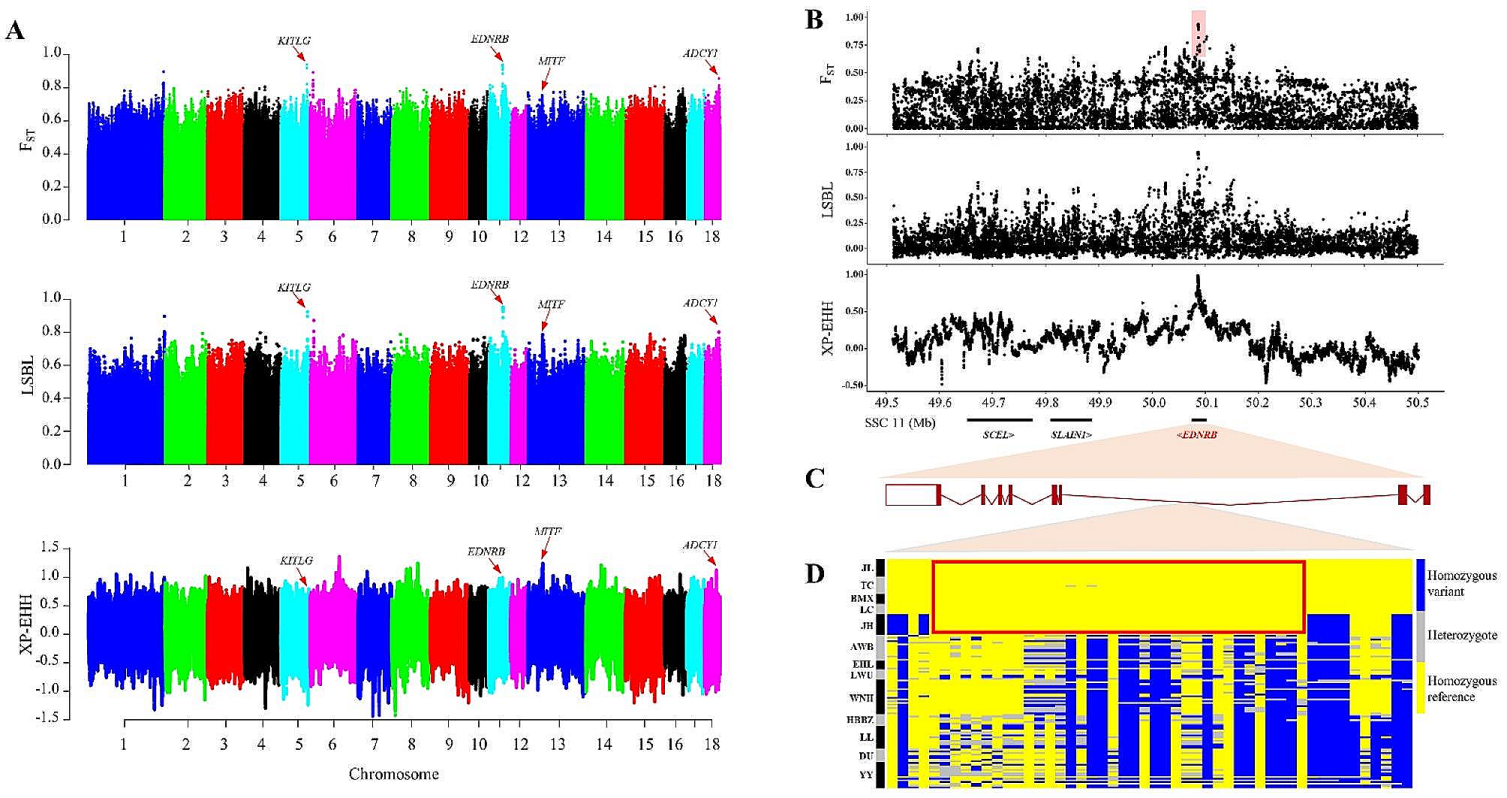
Genomic regions with strong selective sweep signals for two-end black (TEB) coat color. **A**: Manhattan plot of the genome-wide distribution of Fst, LSBL, and XP-EHH value, respectively. **B**: Genome scans along the *EDNRB* region. **C**: The genome structure of the porcine *EDNRB* gene. **D**: Haplotypes at SSC11: 50,086,427 − 50,088,340 region containing the most significant signal. Major and minor alleles are labeled in yellow and blue, respectively

Coat color is a remarkable morphologic feature for breed standard. Jianli pig is a typical two-end black (TEB) colored breed in central China, but the genetic mechanism of its coat color remains unclear. To uncover genomic signals of positive selection for the TEB coat color phenotype, we performed three methods: Fst, LSBL and XP-EHH (Fig. 4A). A region of SSC11, located in the second intron of the *EDNRB* gene, has the most significant Fst and LSBL values (Fig. 4B, 4C). In this significant selection signal, we found a region of 1.9 kb consisting of 36 SNPs, located at SSC11: 50,086,427 − 50,088,340, which was almost fixed in all TEB pig breeds (Fig. 4D).

### *EDNRB* haplotypes in pigs with diverse pigmentation patterns

**Fig. 5:**
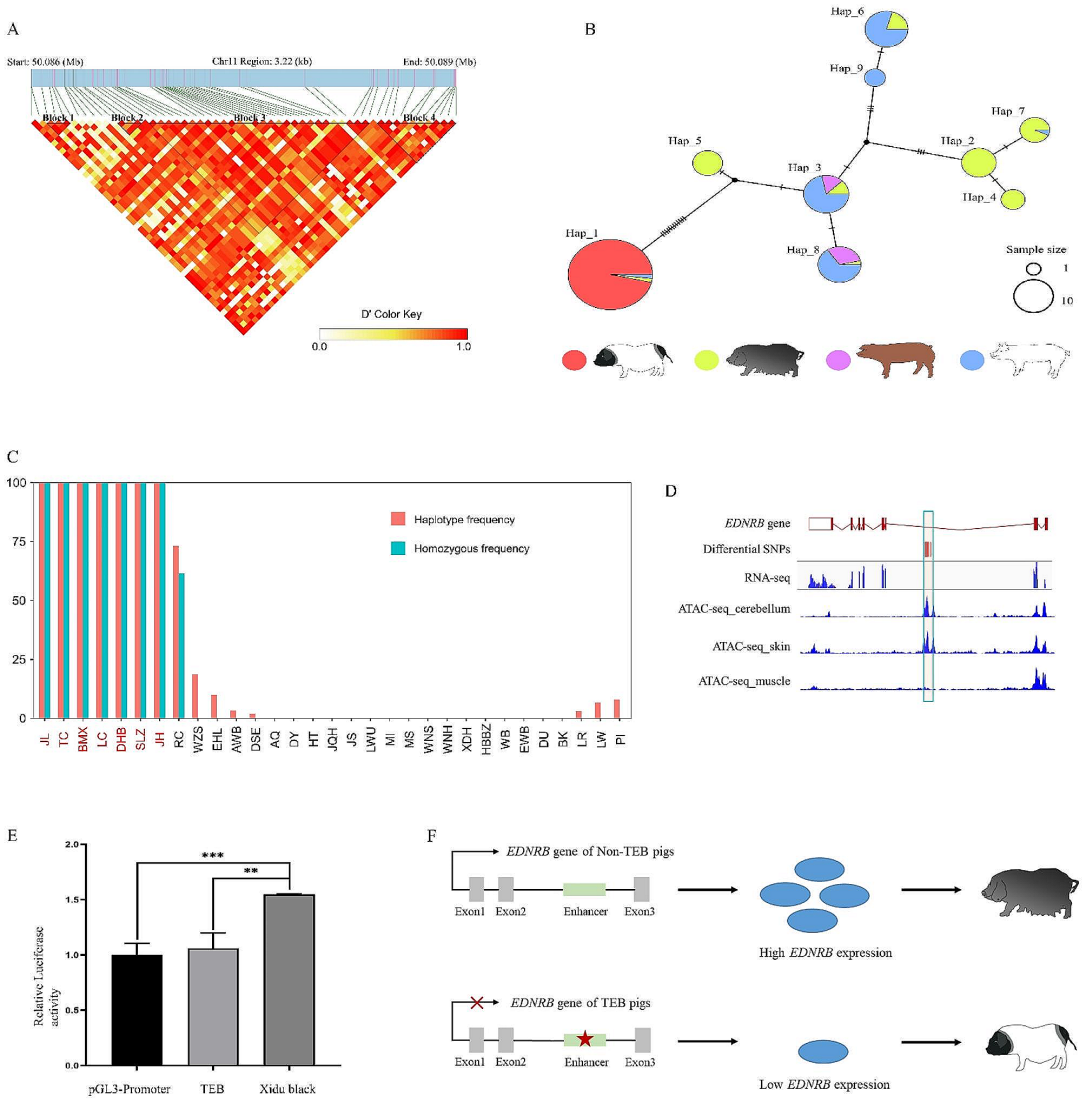
*EDNRB* haplotypes in pigs with diverse pigmentation patterns and candidate causative mutation for the two-end‐black coat color phenotype in pigs. **A**: Haplotype block analysis at the most significant signal from 50.086 to 50.089 Mb on SSC11. **B**: Network of *EDNRB* haplotypes with frequencies greater than two. The haplotypes were defined from 25 SNPs within the *EDNRB* region of 1.2 kb. **C**: Frequencies of the 1.2‐kb *EDNRB* haplotype in TEB and non-TEB color pigs. **D**: Genome browser view of the differential ATAC-seq peak in the *EDNRB* gene. The blue box indicates that significantly selected SNPS are located within the ATAC-seq peak region of the skin, but not in the ATAC-seq of muscle tissue. **E**: Relative luciferase activity comparison between the 1.2‐kb *EDNRB* haplotype of TEB and non-TEB color pigs in the porcine kidney cell line, PK15 cells. The 1.2‐kb *EDNRB* haplotype regions of Jinhua pigs and Xidu black pigs were cloned into pGL3-Promoter vectors. pGL3-Promoter was used as controls. The bars display mean ± SD (N = 3 technical replicates). **F**: A working model showing how the 1.2‐kb *EDNRB* haplotype affects the TEB coat color in pigs

*EDNRB* gene stands out as an extreme LSBL and Fst outlier in the genome scan of TEB and no-TEB coat color pig breeds. We subsequently focused our attention on this gene. Initially, we employed LDBlockShow software for linkage disequilibrium analysis and constructed haplotype blocks for the Chr11: 50.086–50.089 Mb under the default parameters (Fig. 5A). As a result, we found that block 3 (Chr11: 50,086,815–50,088,340), containing 25 SNPs, overlapped with the strongest selection signal region. Then, a haplotype network of these 25 SNPs was further constructed by using 9 common haplotypes with a frequency greater than 2 in all pig breeds (Fig. 5B). Interestingly, TEB pigs only displayed one homozygous haplotype (hap_1), while this haplotype is a heterozygous carrier in one AWB pig, one EHL pig, and two LW pigs. We further investigated the distribution frequencies of the 25-SNPs *EDNRB* haplotypes in 549 individuals from 32 Chinese and European breeds (Fig. 5C, Table S6). Haplotype I was only found in seven TEB pig breeds, three black pig breeds (EHL, AWB, and DES), two white pig breeds (LR and LW) and three black spots breeds (RC, WZS and PI). Of note, the homozygous haplotype of haplotype I was solely present in the seven TEB-colored breeds, and one white breed (Rongchang) with black spots on eyes. Considering the phenotypic changes and related gene functions, haplotype I (TEB-haplotype) of the *EDNRB* gene may regulate the formation of TEB coat color.

To uncover the molecular mechanism of the TEB phenotype, we download six RNA-seq data from a public database (NCBI Accession: PRJNA516660) for black and white skin of three two-year-old Bama Xiang pigs (a kind of TEB breed). The result showed no difference in the expression of *EDNRB* gene in black and white skin tissue of Bama Xiang pigs. Moreover, we did not find alternative transcripts in the black and white skin tissues of Bama Xiang pigs at the *EDNRB* locus, which is consistent with the RNA-seq result as visualized by the IGV software (Figure. S2). To detect whether this haplotype is located in the functional regulatory region, we used publicly available ATAC-seq data from different tissues and found that there were peaks in skin and cerebellum tissues (Fig. 5D). This result suggests that this haplotype may play a regulatory role. We compared the activity of this TEB-haplotype with that of the black-haplotype by designing a luciferase experiment in PK15 cells. Luciferase reporter assays revealed that the 25-SNPs region in Black pig has enhancing functions, and the TEB-haplotype down-regulates the expression of the reporter gene compared to the Black pig (Fig. 5E). Thus our results suggested that this highly selected haplotype of cis-regulatory element causes relatively lower expression of the *EDNRB*, leading to low melanin formation in melanocytes (Fig. 5F).

## Discussion

The Jianli pig is a well-known indigenous breed in China, characterized by its two-end black coloration, early maturity, good meat quality and strong disease resistance. However, its genomic diversity, population structure, and selective signals have not been elucidated yet. In this study, whole genome resequencing was performed on 30 Jianli pigs, within a broader context of 153 individuals representing 13 diverse breeds. The results showed that the division of genetic diversity was consistent with its geographical distribution. According to NJ-tree, the relationships between Jianli and Tongcheng pigs were closer than those of other breeds; this result was also confirmed using pairwise Fst and PCA. Jianli and Tongcheng pigs were both from central China, and both have the two-end black coat color, which was once called “Huazhong two-end black pig”. However, due to the differences in performance between each other, it was divided into two independent breeds [3]. From the perspective of structure, when K = 11, Jianli and Tongcheng pigs formed an independent lineage while Luchuan and Bamaxiang pig were still together, indicating that the genetic distance between Jianli pigs and Tongcheng pigs was greater than that between Luchuan pigs and Bama pigs.

Genetic diversity is an important index to measure the degree of population variation and the status of germplasm resources [24]. Jianli pigs had Ho of 0.21 and He of 0.22, which were comparable with those of most Chinese indigenous breeds. The analysis of runs of homozygosity (ROHs) can be used to address major concerns in conservation genetics, including inbreeding and population demography [25, 26]. Currently, the inbreeding coefficient based on ROH (F_ROH_) is considered one of the most powerful approaches to detect inbreeding [18, 27]. In our study, the average inbreeding coefficient based on the ROH of the Jianli pig was 0.01. Compared with other breeds, Jianli pigs have a lower inbreeding coefficient, and the total number and length of ROH are smaller, indicating that the Jianli breed is still rich in genetic diversity in the context of its neighboring breeds despite the recent decline of its population size. The inbreeding coefficient of Western pigs is higher than that of Chinese pigs, which is probably caused by the strict breeding programs that caused inbreeding during the formation of modern Western pigs.

To reveal the selection signatures of the Jianli pigs during domestication and breeding, we further compared the genomic signatures of Jianli pigs with Asian wild boar pigs using Fst and the XP-EHH methods. A total of 382 selection regions were detected, and 445 candidate genes were further identified within these regions. Functional enrichment analysis indicated that these genes may play important roles in immunoglobulin production, melanocyte differentiation, fibroblast proliferation and male infertility pathways.

We detected a list of genes under selection that showed associations with meat quality, such as *CREBBP*, *ADCY9*, *EEPD1* and *HDAC9*. *CREBBP* and *ADCY9*, which were involved in cAMP signaling, were also detected in the selection region for meat quality in the Anhui autochthonous pig population [28]. Previous GWAS and post-GWAS studies found that *CREBBP* and *ADCY9* are located within regions that were significantly associated with meat pH [29]. The *EEPD1* gene mainly plays a role in repairing stressed replication forks during DNA damage [30]. Studies have shown that the *EEPD1* gene may play a role in fat deposition in cattle [31] and pigs [32]. *HDAC9* could negatively regulate muscle differentiation by inhibiting the transcriptional activity of MEF2 [33]. GWAS and RNA-seq analysis showed that *HDAC9* plays an important role in the loin muscle area of Beijing black pigs [34].

Several candidate genes relating to reproduction were also detected, including *ESR1*, and *FANCA*. The *ESR1* gene, which was associated with litter size in pigs [35], was the most promising functional gene to account for excellent reproductive performance in pigs. The *FANCA* gene, which was also detected in the selection region for reproduction in Anqing six-end-white pigs [36], could affect female fertility in human subjects and mice [37]. Jianli pigs have a higher reproductive capacity compared with the Asian wild boar pigs. Correspondingly, the *ESR1* and *FANCA* genes showed high evidence of positive selection in our study. It is worth mentioning that we found three genes related to coat color: *EDNRB*, *MITF* and *MC1R*. *EDNRB* is an important gene responsible for the TEB phenotype in Chinese indigenous pigs except for Jinhua pig [1, 4, 38]. Previous studies have demonstrated that *MITF* variants were associated with different coat colors in numerous animal species, including cattle [39, 40], horses [41], sheep [42], ducks [43] and pigs [44]. The *MC1R* gene has been shown to be associated with coat color in cattle [45, 46], sheep [47],pigs [48], chickens [49].

Jianli pig is a typical TEB colored breed in China, but the genetic mechanism of its coat color remains unclear. Thus, to fine map the genomic region for the TEB coat color phenotype, we performed three signature selection methods between the TEB coat color and no-TEB coat color pig breeds. The results showed that one region of SSC11, located in the second intron of the *EDNRB* gene had strong signatures of positive selection. *EDNRB* is a pigmentation gene that has been highlighted in previous studies as a candidate gene for the TEB phenotype [1, 5, 38]. Our data provide compelling evidence that *EDNRB* is a promising candidate gene for the TEB phenotype in Chinese pigs. Interestingly, we found a haplotype block (Chr11: 50,086,815–50,088,340) containing 25 SNPs in the strongest selection signal, showing a homozygous haplotype in all TEB pigs. To investigate the distribution frequency of these 25-SNPs *EDNRB* haplotypes, we further expanded the population size and found once more that all TEB-colored breeds exhibit one homozygous haplotype in 549 individuals from 32 Chinese and European breeds. Previous studies have found that, contrary to expectations, *EDNRB* is the gene responsible for the TEB phenotype in Chinese indigenous pigs except for Jinhua pigs [1]. In this study, the 25-SNPs homozygous haplotype was found in all TEB pig breeds, including Jinhua pig, indicating that this haplotype of the *EDNRB* gene may promote the formation of TEB coat color.

We next focused on this specific haplotype block. Since this haplotype was located in the intron region, introns can affect gene expression by changing the form of alternative splicing to produce different transcripts [50]. Therefore, we examined the transcripts of black and white skin tissues of Bama Xiang pigs at the *EDNRB* locus by RNA-seq, but no difference was found between the transcripts of black and white skin tissues. On the other hand, regulatory elements such as enhancers in the intron region can directly regulate gene transcription [51]. Therefore, we hypothesized that the haplotypes harboring these 25 SNPs were able to regulate the coat color by altering enhancer function. To check whether this haplotype was located in the regulatory element, we downloaded ATAC-seq data from different tissues and found that this haplotype was indeed located in the peak of ATAC-seq data in skin tissue. Further luciferase reporter assays revealed that the 25-SNPs region has enhancing functions, and the TEB-haplotype down-regulates the expression of the reporter gene compared to Black pigs. The extent to which non-coding variations contribute to phenotypic variations in animal domestication was largely unknown. Only a few noncoding mutations have been identified, such as an intronic mutation of IGF2 influencing muscle development [52] and a 1.2 kb-haplotype mutation in the intron of the BMPR1B gene was a causal candidate for pig prolificacy [53]. In this study, we further confirmed that the *EDNRB* gene is a candidate gene for TEB color phenotype, including Jinhua pigs, and the haplotype harboring 25 SNPs was a strong candidate causative mutation to regulate the TEB coat color phenotype by altering enhancer function.

## Conclusions

To our knowledge, this is the first study to characterize the genetic variation, phylogenetic relationships, population structure, and selection signatures of the Jianli pigs. The characterization of population structure and genomic diversity will provide directions for genetic assessment and formulation of reasonable breeding strategies for Jianli pigs. Moreover, we identified a series of candidate genes that may have important effects on the meat quality, reproduction, and coat color traits of this breed. In addition, we confirmed a 25 SNPs-haplotype in the intron of the *EDNRB* was a strong candidate causative mutation responsible for the two-end black coat color in pigs. Our work highlights the importance of non-coding variants in shaping phenotypic differences in pigs and provide novel insights into the coat color genetics of pigs.

### Electronic supplementary material

Below is the link to the electronic supplementary material.

**Supplementary Material 1**: **Table S1**: Inbreeding coefficient of each breed. **Table S2**: Potential candidate regions detected by Fst (Jianli VS Asian wild boar). **Table S3**: Potential candidate regions detected by XP-EHH (Jianli VS Asian wild boar). **Table S4**: Overlapping candidate regions identified by the two methods. **Table S5**: Gene annotation of selected regions detected by both methods. **Table S6**: The frequencies of the 25-SNPs EDNRB haplotypes in 549 individuals from 32 breeds

**Supplementary Material 2**: **Figure. S1**: The Gene Ontology (GO) terms and KEGG pathways of the 451 candidate genes in Jianli pigs. **Figure. S2**: EDNRB transcripts in the skin of Bama Xiang pigs

## Data Availability

Sequencing reads of Jianli and Jinhua pigs have been submitted to (National Center for Biotechnology Information) NCBI with accession number PRJNA1025849. The datasets analysed in this study were downloaded from the NCBI Sequence Read Archive (http://www.ncbi.nlm.nih.gov/sra/) under project PRJNA213179, PRJNA488960, PRJNA524263, and PRJNA260763.

## Abbreviations

He: Expected heterozygosity
Ho: Observed heterozygosity
IBS: Identical-by-state
PCA: Principal component analysis
ROH: Runs of homozygosity
SNP: Single nucleotide polymorphism
TEB: Two-end black
WGS: Whole-genome sequencing
