# Supplementary material for: Genome-wide detection of selection signatures in Jianli pigs reveals novel cis-regulatory haplotype in EDNRB associated with two-end black coat color
Source: BMC Genomics. 2024 Jan 2;25:23. doi: 10.1186/s12864-023-09943-9 (PMC10763394; doi:10.1186/s12864-023-09943-9)

Genome-wide detection of selection signatures in Jianli pigs reveals novel cis-regulatory haplotype in *EDNRB* associated with two-end black coat color

Figure.S1 The Gene Ontology (GO) terms and KEGG pathways of the 451 candidate genes in Jianli pigs.


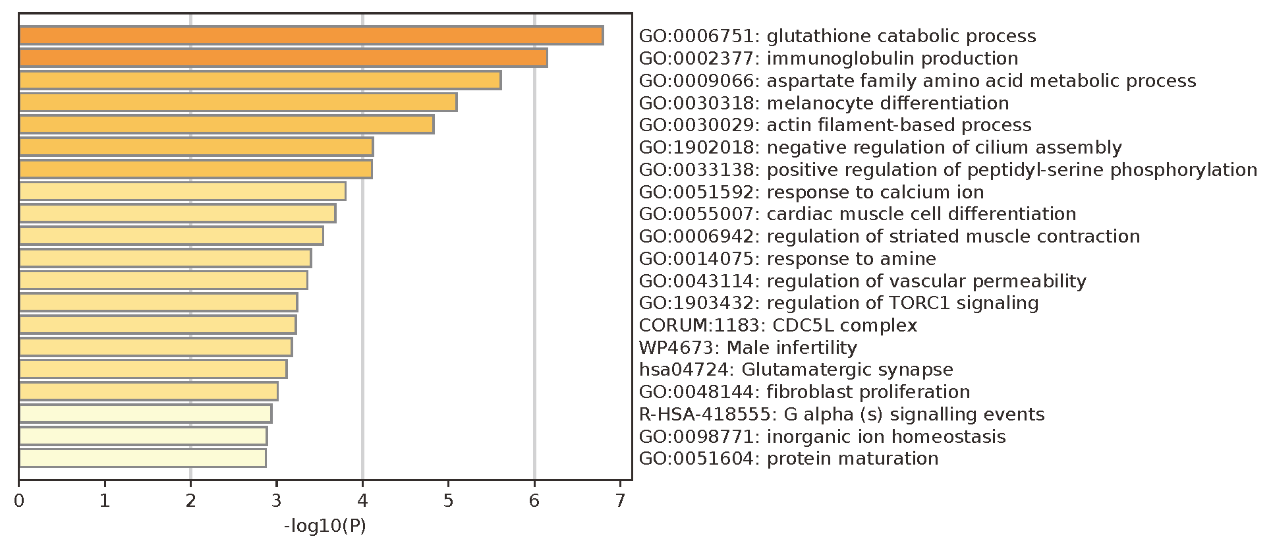


Figure.S2 *EDNRB* transcripts in the skin of Bama Xiang pigs.


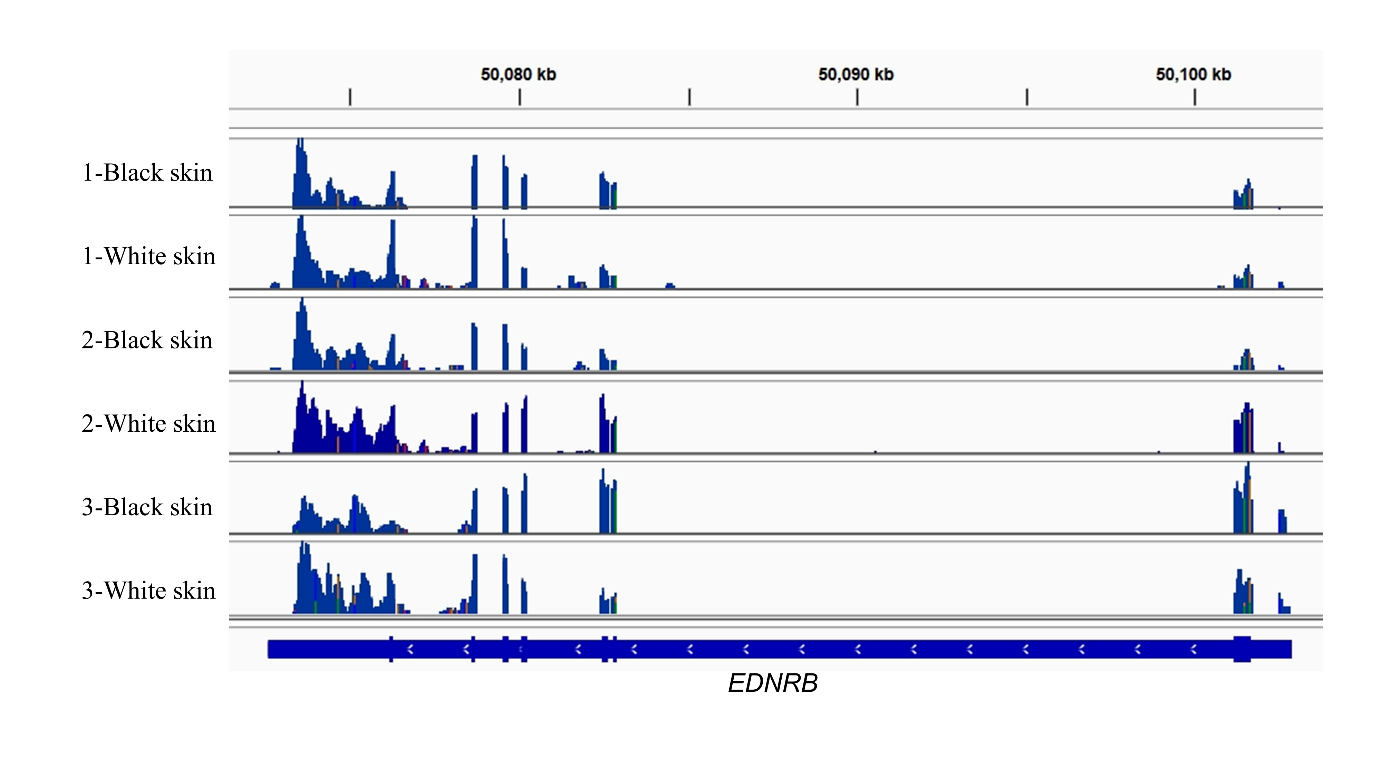

Supplement: Supplementary file 2 — Supplementary Material 2: Figure. S1: The Gene Ontology (GO) terms and KEGG pathways of the 451 candidate genes in Jianli pigs. Figure. S2: EDNRB transcripts in the skin of Bama Xiang pigs [file 12864_2023_9943_MOESM2_ESM.docx]
